# A new transmission map of HIV-1 CRF07_BC in China: analysis of sequences from 12 provinces over a decade

**DOI:** 10.1186/1742-4690-9-S2-P139

**Published:** 2012-09-13

**Authors:** M Zhefeng, L Jingyun, Z Ping, X Jianqing, Z Xiaoyan

**Affiliations:** 1 Shanghai Public Health Clinical Center, Shanghai, China; 2 Academy of Military Medical Science, Beijing, China; 3 Shanghai Municipal Centers for Disease Control and Prevention, Shanghai, China

## Background

HIV-1 CRF07_BC descended from subtypes B’ and C represents one of the most prevalent HIV-1 strains in Asia and has been devastating in IDUs for more than a decade in China. Recently, several groups made their effort to address its origin and migration pattern, and had generated several enlightening conclusions, however, the limited available sequences may have restrained the precise depiction of global migration pattern of CRF07_BC. In this study, epidemic evidences and sequences analysis was combined to investigate the transmission linkages among 12 CRF07_BC epidemic regions in China and their tMRCA(time of most recently common ancestor).

## Methods

138 of 769 sequences covered 12 provinces (including newly generated 45 sequences) was identified as independent sequences and used for subsequent phylogeographic tree analysis, Bayes Factor test. Almost all Chinese literatures on early HIV-1 epidemic history are reviewed to confirm the results of sequences analysis.

## Results

The initial transmission occurred in Guangxi (eastern neighbor to Yunnan) in 1994 and Xinjiang (northwest) in 1995 and Sichuan (northern neighbor to Yunnan) in 1996 after CRF07_BC generated in Yunnan around 1993. The subsequent transmissions occurred from Yunnan to Liaoning (northeast) in 1997 and Jiangsu in 1998. Interestingly, after the initial epidemic regions, including Guangxi, Xinjiang and Sichuan, served as secondary epicenters for further spreading, triggered further transmission into Gansu, Ningxia, Qinghai, Beijing and Hunan during 1999-2001. These analyzed results are in accordance with early epidemic investigations of HIV-1.

## Conclusion

Our data indicated that both the origin site and secondary epicenters played important roles in the dynamic migration of CRF07_BC in China, and both drug traffic and population migration (which largely occurred from west to east and from other regions to capital city during last two decades) may have significantly contributed to the complicated transmission pattern of CRF07_BC.

